# Compound Heterozygous Hemoglobin E-Beta (HbE-β)-Thalassemia Presenting With Chipmunk or Rodent Facies, and a Severe Thalassemia Major Phenotype

**DOI:** 10.7759/cureus.103687

**Published:** 2026-02-15

**Authors:** Anupam Dutta, Ankita Banik, Dipankar Das, Taniya S Dutta

**Affiliations:** 1 Department of General Medicine, Assam Medical College and Hospital, Dibrugarh, IND; 2 Department of Medicine, Assam Medical College and Hospital, Dibrugarh, IND; 3 Department of Pediatrics, Assam Medical College and Hospital, Dibrugarh, IND

**Keywords:** compound heterozygosity, extramedullary hematopoiesis, genotype-phenotype association, hbe–β thalassemia, rodent facies

## Abstract

Hemoglobin E-beta (HbE-β)-thalassemia is a common compound hemoglobinopathy in South and Southeast Asia and is marked by considerable clinical heterogeneity, ranging from mild anemia to a severe, transfusion-dependent condition resembling β-thalassemia major (β-TM). The severity of disease is largely determined by the nature of the associated β-thalassemia mutation, modifiers of fetal hemoglobin, and access to early diagnosis and regular treatment. Severe skeletal deformities are now uncommon in the era of structured transfusion programs, but continue to be encountered in regions with delayed diagnosis and inadequate care.

We report a 20-year-old female from Northeast India who presented with progressive facial disfigurement, dental malocclusion, growth failure, and delayed puberty. She had a history of long-standing anemia treated intermittently with blood transfusions and no prior iron chelation. Clinical examination revealed classical craniofacial abnormalities, including frontal bossing, prominent malar eminences, and marked maxillary hypertrophy, producing the characteristic chipmunk or rodent facies. Radiological evaluation of the skull demonstrated widening of the diploë with a hair-on-end appearance, consistent with chronic marrow hyperplasia and extramedullary hematopoiesis. Molecular analysis confirmed compound heterozygosity for HbE (HBB:c.79G>A) and a severe β-thalassemia splice-site mutation, IVS-I-5 (G>C) (HBB:c.92+5G>C). Despite the HbE-β-thalassemia genotype, the patient exhibited a phenotype indistinguishable from classical β-TM, including severe anemia, stunted growth, primary amenorrhea, and advanced skeletal deformity.

This case highlights the severe end of the HbE-β-thalassemia spectrum and underscores that compound heterozygous states may present with a β-TM-like phenotype. Early diagnosis, regular transfusion-chelation therapy, and genotype-phenotype-guided management are essential to prevent irreversible skeletal and endocrine complications.

## Introduction

Hemoglobin E-beta (HbE-β)-thalassemia is one of the most prevalent compound hemoglobinopathies in Southeast Asia, resulting from the co-inheritance of the structural variant HbE (HBB:c.79G>A) on one allele and a β-thalassemia mutation on the other [1]. The clinical spectrum ranges widely, from transfusion-independent mild anemia to severe transfusion-dependent disease mimicking classical β-TM [2]. The phenotypic variability is influenced by the nature of the β-thalassemia mutation, co-inherited α-thalassemia, fetal hemoglobin modifiers, and genetic/environmental factors [3].

Patients with severe genotypes, particularly when the β-mutation is severe or affects splicing (such as IVS-I- 5 G>C), often exhibit ineffective erythropoiesis, profound anemia, and transfusion dependence similar to β-TM [4]. Chronic marrow hyperplasia and expanded erythropoietic activity may produce skeletal deformities, including frontal bossing, malar prominence, and maxillary overgrowth, resulting in the classic “thalassemic facies” or rodent facies [5]. Such craniofacial remodelling not only leads to functional impairment of occlusion and speech but also causes a substantial psychosocial burden.

Although routine neonatal screening and early transfusion-chelation programs have reduced the incidence of advanced deformities, resource-limited settings still report late-diagnosed patients with severe skeletal and endocrinological manifestations. Recognition of severe phenotypes in HbE-β-thalassemia remains important, particularly when the presenting picture is indistinguishable from β-thalassemia major.

We describe a young female with compound heterozygous mutations, HbE (HBB:c.79G>A) and IVS-I-5 (G>C) (HBB:c.92+5G>C), who presented with growth failure, delayed puberty, and marked maxillofacial hypertrophy, phenotypically resembling severe β-TM. This case emphasizes the importance of timely diagnosis, genotype-phenotype correlation, and multidisciplinary management in HbE-β-thalassemia compound heterozygous hemoglobinopathy with severe skeletal disfigurement.

## Case presentation

A 20-year-old female from Northeast India presented to the outpatient clinic with a gradually progressive alteration in facial appearance noted since early adolescence. Over the preceding two years, the deformity had become more pronounced, characterized by increasing prominence of the maxillary region and malar bones with relative mandibular retrusion, resulting in a conspicuous change in facial contour. She also reported significant dental crowding. These structural changes were associated with functional impairment, including difficulty chewing solid food, prolonged mastication, and occasional choking episodes attributable to malocclusion. Additionally, she described progressive difficulty in speech articulation, particularly involving consonants requiring precise labiodental and dental movements, leading to increasing social discomfort. The patient reported intermittent facial pain and a sensation of heaviness during chewing or prolonged speech. There was no history of headache, visual disturbance, nasal obstruction, or hearing impairment.

**Figure 1:**
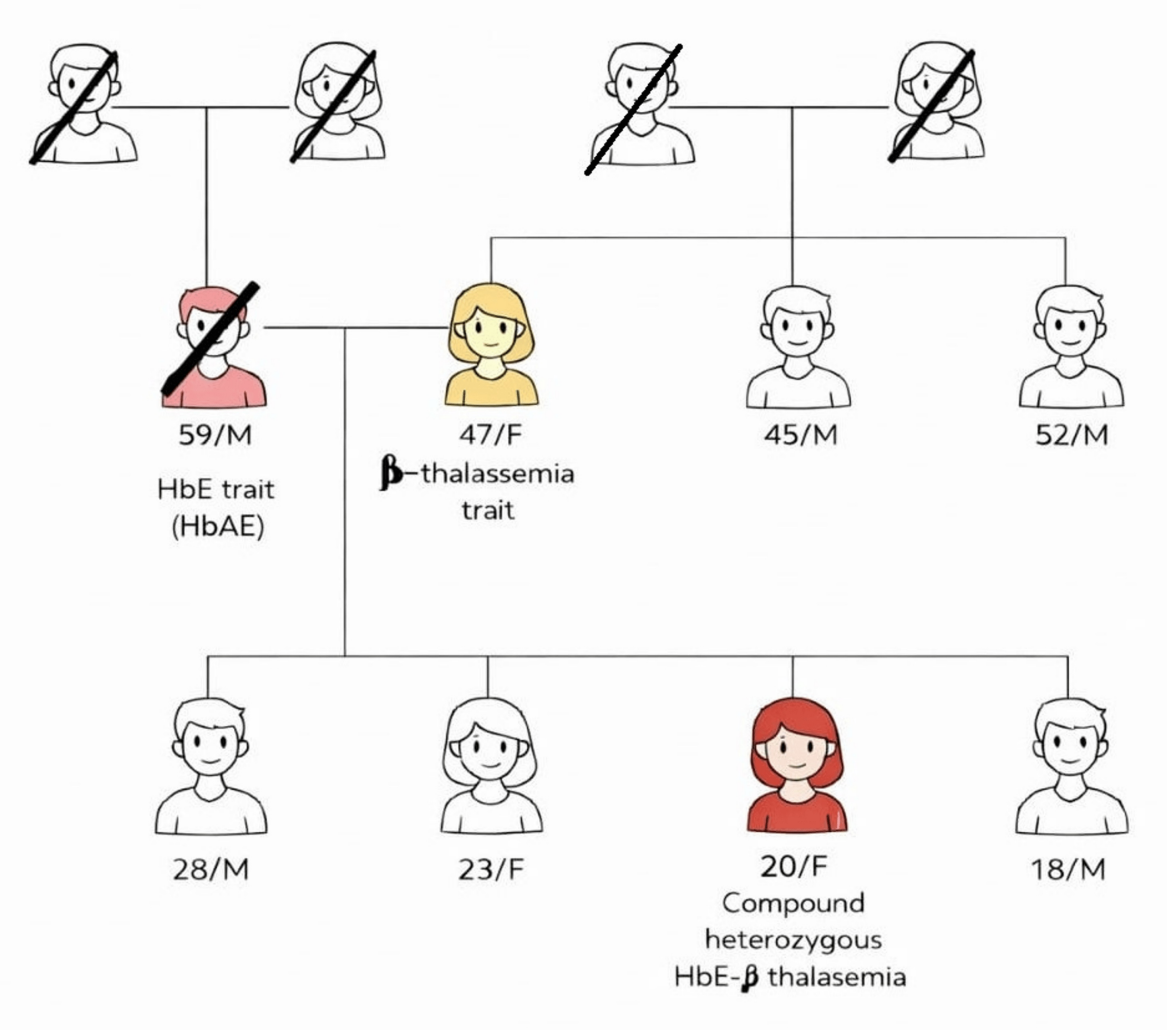
Family tree of a 20-year-old compound heterozygous case of HbE-β-thalassemia. Her father (deceased) had HbE trait, and her mother has β-thalassemia. Two of her brothers (28 and 18-year-olds) and one sister (23-year-old) are apparently healthy. Further genetic testing may reveal a trait in the siblings.

Family history from the elder brother revealed that the patient was born to non-consanguineous parents, although no documentation was available. Her deceased father was a carrier of the HbE trait, while her mother is a carrier of the β-thalassemia trait (no genetic confirmation was available). She has three siblings, all of whom are reportedly asymptomatic, with no history of anaemia, transfusion dependence, or other haematological disorders (Figure 1).

She had primary amenorrhea and had not developed secondary sexual characteristics, which is suggestive of delayed pubertal development. At 14 years of age, she was hospitalized for generalized weakness associated with recurrent dragging pain in the left hypochondrium. The pain had persisted intermittently over several years and was attributed to progressive splenomegaly, for which she eventually underwent splenectomy in February 2025. During that admission, peripheral blood smear examination and hemoglobin electrophoresis were suggestive of thalassemia; however, the patient was subsequently lost to structured follow-up. No regular transfusion protocol or iron chelation therapy was initiated. In the ensuing years, she received only sporadic blood transfusions during periods of symptomatic anemia.

**Figure 2:**
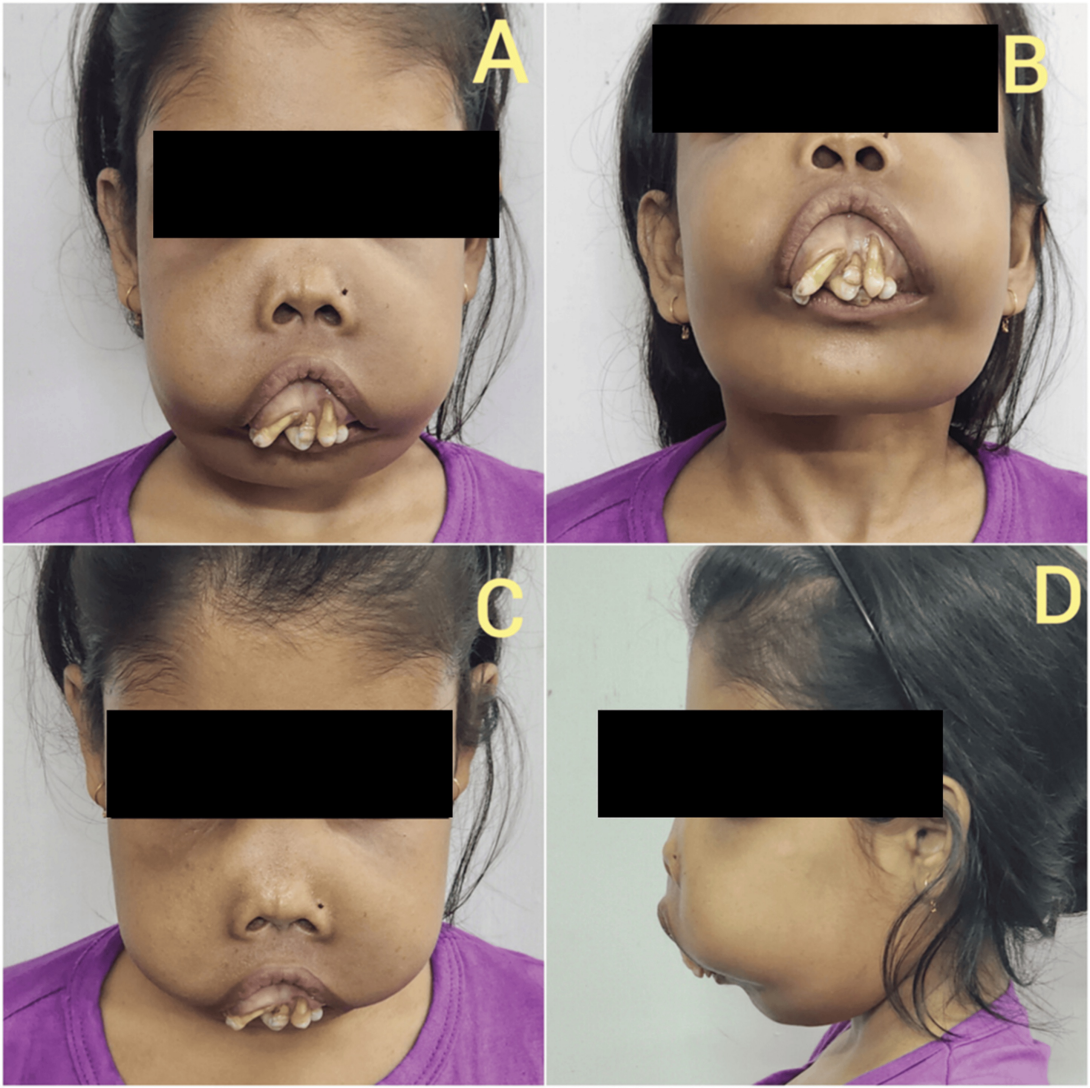
Classical thalassemic (chipmunk or rodent) facies. A. Frontal bossing, malar prominence, and advanced maxillary hypertrophy with forward-projecting incisors and severe arch crowding; B. Malar outgrowth and dental malalignment; C. Flattening of the nasal bridge, hypertrophied maxillary bone and crowding of the teeth; D. Lateral view of the face.

On physical examination, the patient exhibited classical thalassemic facies, characterized by prominent frontal bossing, widened and elevated malar eminences, and marked maxillary hypertrophy with anterior protrusion of the upper jaw. The maxillary overgrowth resulted in forward-projecting upper incisors, severe dental crowding, and malocclusion, contributing to the characteristic chipmunk or rodent facies (Figure 2). The nasal bridge appeared flattened, and the overall facial profile was convex due to maxillary prominence. She had significant pallor, mild icterus, and features of chronic undernutrition. There was no history or evidence of prior corrective maxillofacial surgery.

Systemic examination revealed no hepatosplenomegaly (post-splenectomy), no lymphadenopathy, and no clinical signs of cardiac failure. Cardiovascular, respiratory, and neurological examinations were otherwise unremarkable.

Anthropometric assessment revealed marked growth failure and undernutrition. The patient had a height of 135 cm and a weight of 30 kg, both far below the expected norms for age and sex, consistent with severe short stature and low body mass. Her body mass index (BMI) (weight in kilograms divided by the square of height in meters) was 16.5 kg/m², indicating chronic undernutrition. Mid-upper arm circumference measured 18 cm, further supporting reduced muscle mass and protein-energy malnutrition. Body proportions were abnormal, with an arm span of 118 cm, significantly shorter than height, suggesting disproportionate skeletal growth. Segmental measurements showed an upper segment length of 63 cm and a lower segment length of 72 cm, yielding an upper-to-lower segment ratio of 0.87, consistent with long-standing growth disturbance affecting the axial and appendicular skeleton.

Central and peripheral anthropometric indices demonstrated additional abnormalities. Waist circumference was 66 cm and hip circumference was 56 cm, resulting in an elevated waist-hip ratio of 1.2, reflecting reduced gluteofemoral fat and altered body fat distribution. A waist-hip ratio >1.0 in a female is unusually high and typically indicates central obesity, but in this chronically undernourished patient, this finding is paradoxical. It is probably due to hepatosplenomegaly and severe muscle wasting in the hips-gluteal region rather than true central fat accumulation. Chest circumference measured 80 cm, consistent with a narrow thoracic cage often seen in chronically anemic states. Triceps skinfold thickness was 19 mm, suggesting relatively preserved subcutaneous fat despite overall low body mass, a pattern described in chronic disease-associated malnutrition. Collectively, these anthropometric findings indicate severe stunting, disproportionate skeletal growth, and chronic nutritional compromise, likely resulting from long- standing anemia, ineffective erythropoiesis, delayed puberty, and inadequate disease control in transfusion- dependent thalassemia (TDT) (Table 1).

**Table 1:** Anthropometric measurements of our patient.

Parameters	Measurements (Unit)
Height	135 cm
Weight	30 Kg
Body Mass Index	16.5 kg/m^2^
Mid Upper Arm Circumference	18 cm
Waist Circumference	66 cm
Hip Circumference	56 cm
Waist-Hip-Ratio	1.2 : 1
Arm Span	118 cm
Chest Circumference	80 cm
Upper Segment	63 cm
Lower Segment	72 cm
Triceo Fold Thickness	19 mm

Laboratory evaluation demonstrated moderate anemia with a hemoglobin level of 7.9 g/dL, an elevated reticulocyte count (9.5%), and high-performance liquid chromatography findings consistent with an HbE trait. Indirect hyperbilirubinemia was noted (1.98 mg/dL), along with elevated serum ferritin levels (940 ng/mL). Serology for viral infections like Hepatitis B, Hepatitis C and HIV was negative. Further genetic evaluation for thalassemia beta mutation analysis using polymerase chain reaction (PCR) sequencing confirmed compound heterozygosity for HbE (HBB:c.79G>A) and β-thalassemia splice-site mutation IVS-I-5 (G>C) (HBB:c.92+5G>C), establishing the diagnosis of HbE-β-thalassemia with a severe β-thalassemia major-like phenotype (Table 2).

**Table 2:** Laboratory parameters of this patient during hospital admission. PCV: packed cell volume; MCV: mean corpuscular volume; MCH: mean corpuscular hemoglobin; MCHC: mean corpuscular hemoglobin concentration; TIBC: total iron binding capacity.

Parameters	Patient Values (Unit)	Reference Levels (Unit) [6]
Hemogram		
Hemoglobin	7.9	12-16 gm/dl
Total Count	18800	4000-11000 cells/uL
Differential Count		
Neutrophils	38	40-75%
Lymphocytes	54	20-45%
Monocytes	6	2-8%
Eosinophils	2	1-6%
Basophils	-	<1%
Platelet Count	629000	1,50000-4,50000/uL
PCV	22.7	36-46%
MCV	72	80-96 fL
MCH	25.2	27-32 pg
MCHC	34.8	32-36 g/dL
Reticulocyte Count	9.5%	0.5-2.5%
Iron Profile		
Serum Iron	219	37-170 ug/dl
Serum Ferritin	940	6.24-137 ng/ml
TIBC	218	265-497 ug/dl

Other laboratory tests include liver function test, kidney function test and genetic testing (Table 3).

**Table 3:** Other laboratory tests. ANA: antinuclear antibody; SGOT: serum glutamic oxaloacetic transaminase; SGPT: serum glutamic pyruvate transaminase; AST: aspartate aminotransferase; ALT: alanine aminotransferase.

Kidney Function Test	Patient Values	Reference Levels (Unit) [6]
Urea	14.97 mg/dl	10-45 mg/dl
Creatinine	0.28 mg/dl	0.3-0.8 mg/dl
Sodium	136 mmol/L	135-145 mmol/L
Potassium	4.08 mmol/L	3.5-5.1 mmol/L
Liver Function Test		
Total Protein	9.26 gm/dL	6.0-8.0 gm/dL
Albumin	4.92 gm/dL	3.5-5.0 gm/dL
Globulin	4.34 gm/dL	2.0-3.5 gm/dL
Total Bilirubin	3.00 mg/dL	0.2-1.2 mg/dL
Indirect Bilirubin	2.00 mg/dl	0.0-1.1 mg/dl
Direct Bilirubin	1.0 mg/dl	0.0-0.30 mg/dL
SGOT (AST)	26 U/L	10-40 U/L
SGPT (A LT)	40 U/L	7-56 U/ L
Alkaline Phosphatase	234 U/L	150-420 U/L

**Figure 3:**
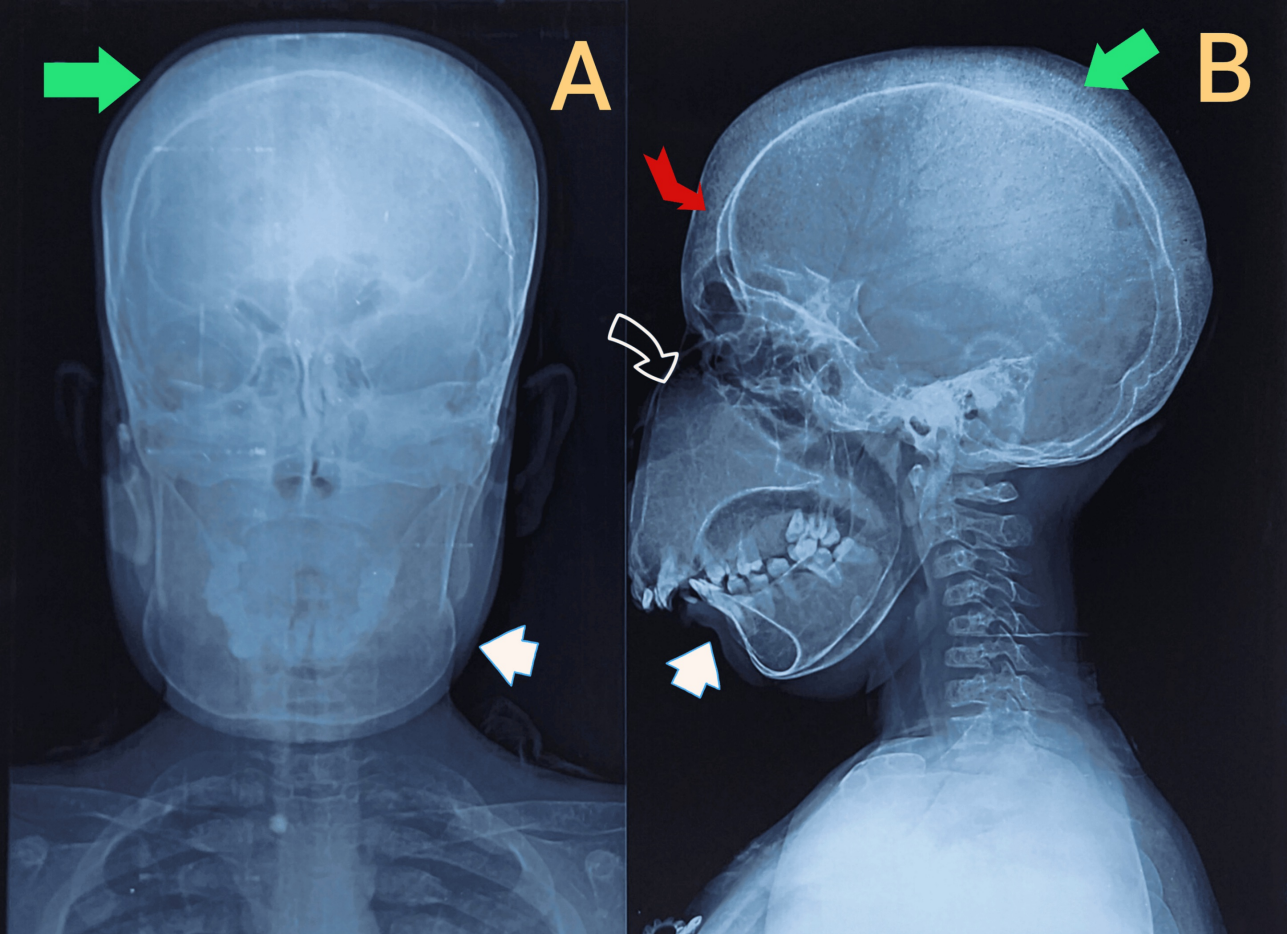
X-ray of skull. A. Anterior-posterior view; B. Lateral view. (black arrow with white border shows maxillary hypertrophy, white arrows show malalignment or malocclusion of teeth, red arrow shows frontal bossing, green arrows show hair-on-end appearance on skull X-ray).

The skull radiographs (frontal and lateral views) show classical skeletal changes associated with chronic ineffective erythropoiesis in severe thalassemia syndromes. There is marked frontal bossing with expansion of the calvarial diploë, accompanied by a distinct hair-on-end appearance, indicating intensified hematopoiesis within the skull bones. The maxillary region demonstrates prominent overgrowth with anterior projection of the upper jaw, consistent with marrow hyperplasia and extramedullary hematopoiesis. This maxillary expansion has resulted in significant dental crowding, protrusion of the incisors, and malocclusion, clearly appreciable in the lateral projection. Together, the craniofacial remodelling, including frontal prominence, widened zygomatic arches, and maxillary hypertrophy, produces the characteristic chipmunk or rodent facies described in literature for poorly transfused or late-diagnosed thalassemia cases. These radiological hallmarks correlate with chronic marrow expansion in response to severe anemia and have been well documented in β-thalassemia and HbE-β-thalassemia, particularly in regions with limited early diagnosis and treatment access (Figure 3).

In view of the severe craniofacial skeletal deformity, associated nutritional compromise, delayed pubertal development, and a history of suboptimal disease control, the patient is currently undergoing multidisciplinary evaluation involving hematology, endocrinology, and maxillofacial surgery services for systemic optimization and planning of staged corrective facial reconstruction. Ultrasonography of the whole abdomen revealed hepatomegaly with associated cholelithiasis, consistent with chronic hemolysis and long-standing hemoglobinopathy. 

## Discussion

HbE-β-thalassemia represents the most common form of severe β-thalassemia in Southeast Asia and the Indian subcontinent and is increasingly recognized as a major contributor to TDT worldwide. It results from compound heterozygosity for a structural hemoglobin variant, HbE (HBB:c.79G>A), and a β-thalassemia mutation on the second allele. The clinical phenotype of HbE-β- thalassemia is remarkably heterogeneous, ranging from mild, non-transfusion-dependent thalassemia (NTDT) to a severe β-TM-like disease requiring lifelong transfusions [5].

Olivieri et al. comprehensively described HbE-β-thalassemia as a clinically diverse disorder, emphasizing that phenotype severity is primarily influenced by the type of associated β-thalassemia mutation, fetal hemoglobin (HbF) modifiers, co-inherited α-thalassemia, and environmental factors [7]. In a large review from South Asia, the authors highlighted that patients with severe β⁰ or splice-site mutations often behave indistinguishably from classical β-TM. Olivieri further elaborated in an Indian cohort that delayed diagnosis and irregular transfusion remain major determinants of skeletal and endocrine complications in resource-constrained regions [7].

Chaudhary et al. reported that among Indian patients, the IVS-I-5 (G>C) mutation is the most prevalent β-thalassemia mutation and is well known to be associated with a severe reduction in β-globin synthesis. When this mutation coexists with HbE in a compound heterozygous state, the resultant imbalance of α and non-α chains leads to marked ineffective erythropoiesis and progressive marrow expansion [8]. Dehury et al. documented a compound heterozygous genotype involving IVS-I-5 with another severe β-thalassemia variant and demonstrated significant clinical severity, including transfusion dependence and splenomegaly [9]. Chaudhary et al. also reported a compound heterozygous β(+)/β(0) genotype involving splice-site mutations, reinforcing the role of IVS-I-5 in generating severe phenotypes [8]. Interestingly, homozygosity for IVS-I-5 may sometimes produce a milder phenotype, as shown by Bohara et al. [10], where unexpectedly mild disease was observed in select patients.

However, when IVS-I-5 occurs in compound heterozygosity with other destabilizing variants or HbE, the disease course is often severe due to compounded transcriptional and post-transcriptional defects. This highlights the critical importance of genotype-phenotype correlation in predicting disease severity.

Wu et al. analyzed a case series of patients with compound heterozygous β-thalassemia mutations and confirmed that those with splice-site and structural hemoglobin variants demonstrated the most aggressive clinical patterns, including early transfusion requirement, growth retardation, and skeletal deformities [11]. Their molecular characterization reinforced that heterogeneous genotypes can converge into uniform severe clinical phenotypes if ineffective erythropoiesis is profound.

India bears a disproportionate burden of HbE-β-thalassemia, particularly in eastern and northeastern states. Satpute et al. confirmed that IVS-I-5 remains the dominant β-thalassemia mutation across western and central India and is strongly linked with transfusion-dependent disease [12]. Mondal et al. documented a high frequency of HbE and IVS-I-5 mutations in large Indian screening programs and noted that many affected individuals remain undiagnosed until late childhood or adolescence. This delayed diagnosis allows unopposed erythroid expansion to drive progressive skeletal remodelling [13]. The craniofacial deformities seen in long-standing HbE-β-thalassemia arise from persistent marrow hyperplasia and extramedullary hematopoiesis, leading to expansion of the diploic of skull bones and the facial skeleton. These include frontal bossing, maxillary hypertrophy, zygomatic prominence, and dental malocclusion, collectively described as chipmunk facies or rodent facies. 

The skeletal manifestations in the present patient-marked maxillary hypertrophy, severe dental crowding, frontal bossing, and hair-on-end appearance-are classic radiological consequences of chronic ineffective erythropoiesis. These findings are fully concordant with the advanced disease phenotypes described in severe compound heterozygous β-thalassemia states. The hair-on-end appearance reflects perpendicular trabecular striations formed due to expansion of active red marrow within the calvarium.

Endocrinopathies are another hallmark of long-standing inadequately treated HbE-β-thalassemia. Delayed puberty, primary amenorrhea, growth failure, and short stature result from chronic anemia, iron overload, pituitary siderosis, and nutritional deficiency. The profound stunting (3.5 feet) and absence of secondary sexual characteristics in the present patient strongly point toward chronic, untreated hypogonadotropic hypogonadism, a late complication well described in severe transfusion-dependent phenotypes. The present case is particularly instructive because the genotype-compound heterozygosity for HbE (HBB:c.79G>A) and IVS-I-5 (G>C) is classically associated with HbE-β-thalassemia. Yet, the patient exhibited a phenotype indistinguishable from classical β-thalassemia major. This reinforces the concept proposed by Olivieri et al. and Bohara et al. that HbE-β-thalassemia should no longer be regarded as uniformly mild or intermediate, but rather as a continuum of disease with potentially severe outcomes [7,10].

From a public health and preventive genetics perspective, population-level mutation mapping has demonstrated that IVS-I-5 and HbE together account for a substantial proportion of severe thalassemia syndromes in India [13]. Verma IC et al. highlighted that regional mutation clustering necessitates targeted premarital and antenatal screening strategies to prevent the birth of affected offspring [14].

## Conclusions

This case highlights a severe and unusual presentation of compound heterozygous HbE-β-thalassemia manifesting with a β-TM-like phenotype, including profound growth failure, delayed puberty, and advanced craniofacial skeletal deformities producing classical chipmunk/rodent facies. Despite a genotype often associated with variable clinical severity, delayed diagnosis, irregular transfusion support, and absence of iron chelation resulted in unchecked ineffective erythropoiesis and irreversible skeletal and endocrine complications. This report underscores the critical importance of early detection, genotype-phenotype correlation, and structured long-term care in patients with HbE-β-thalassemia, particularly in resource-limited settings. A multidisciplinary approach encompassing hematological optimization, endocrine evaluation, nutritional rehabilitation, and staged maxillofacial correction is essential to improve functional outcomes and quality of life in such patients.
